# The Implications of COVID-19 for Early Childhood Education in Ethiopia: Perspectives from Parents and Caregivers

**DOI:** 10.1007/s10643-021-01214-0

**Published:** 2021-05-31

**Authors:** Janice H. Kim, Mesele Araya, Belay Hagos Hailu, Pauline M. Rose, Tassew Woldehanna

**Affiliations:** 1 Faculty of Education, University of Cambridge, 184 Hills Road, Cambridge, CB2 8PQ UK; 2 Department of Economics, Addis Ababa University, Addis Ababa, Ethiopia; 3 Institute of Educational Research, Addis Ababa University, Addis Ababa, Ethiopia

**Keywords:** Early childhood education, Early learning, COVID-19, Parental involvement, Educational inequality

## Abstract

Recent research on the effects of COVID-19 on school closures has mainly focused on primary and secondary education, with extremely limited attention to early childhood education (ECE). To address this gap, we identify the extent to which parents and caregivers with pre-primary school-aged children were engaged in their children’s learning during school closures in Ethiopia. Our focus on Ethiopia is of particular relevance given that ECE provision has expanded dramatically in recent years, aimed at ensuring children are prepared for primary school. Using data collected through a phone survey with 480 parents and caregivers, the results revealed that learning disruption due to COVID-19 school closures is likely to be substantial and will probably widen existing inequalities further. Many poorer households and those where parents or caregivers are not literate, are less likely to have child-oriented learning resources, and home learning activities between parents and children in these households are limited. The study highlights that greater attention needs to be paid to mitigate the threats of COVID-19 on Ethiopia’s recent gains in ECE, to prevent the pandemic from further reinforcing inequalities between children from advantaged and disadvantaged households.

## Introduction

The outbreak of COVID-19, with its associated economic and social challenges, has led to serious consequences for the schooling of children worldwide. A recent study indicates that the interruption of early childhood education (ECE)^1^ programmes could result in potentially large losses in education, health, income, and productivity over their lifetime (Lopez Boo, et al., 2020). However, globally, most attention is currently being paid to the likely adverse effects on children’s learning in primary and secondary schools, with very little attention to ECE. While the immediate, medium- and long-term impacts of COVID-19 on households remain uncertain, there is an urgent need for timely information to help monitor and mitigate the effects of COVID-19 on young children, especially for those who are exposed to particular risks of exclusion and disadvantage.

ECE in Ethiopia has expanded dramatically in recent years with the government’s commitment to providing access to all 6-year-old children, with the intention of expanding ECE gradually to include 4- and 5-year olds. In line with other countries worldwide, schools in Ethiopia were closed due to the pandemic on 16 March 2020. More than 26 million students from over 47,000 schools nationwide were affected by the closures, including 3.2 million young children who had been participating in ECE. However, while primary and secondary have received attention in the government’s COVID-19 response planning (Ministry of Education, 2020), there has not been a clear response strategy for ECE in the light of school closures. Although schools started to re-open in Ethiopia from October 2020, little is known about the extent to which parents and caregivers had been able to support young children’s learning and wellbeing during school closures. This has implications for the type of support that children were likely to need as schools re-opened. Given that school disruptions due to COVID-19 are likely to continue, the analysis also provides lessons for identifying areas that will continue to need attention to ensure households can support their children’s learning at home. These lessons from Ethiopia will also be of relevance to other low-income contexts that are facing similar challenges.

Young children are particularly vulnerable in times of crisis, especially in poor communities. Even before the pandemic, 43% of all children younger than 5 years of age in low- and middle-income countries, were estimated to be at risk of not achieving their developmental potential (Black et al., 2017). Research on the effects of prior pandemics and disasters indicates that there will be both immediate and long-term negative consequences for many children, especially during early childhood, given a clear link between early adversity and later impairments in learning, behaviour, and both physical and mental wellbeing (Shonkoff et al., 2012). The COVID-19 pandemic has the potential to impose more severe constraints on young children’s development through increases in economic instability and food insecurity, heightened stress of caregivers, and decreased health care and social support (Yoshikawa et al., 2020). Unless there is a commitment by governments in lower-income contexts to support coordinated, multisectoral responses, a larger number of children is at risk of facing physical, socioemotional, and cognitive consequences over the entire course of their lives.

In this regard, the current study focuses on the implications of the COVID-19 pandemic on early learning continuity in Ethiopia from the perspectives of parents and caregivers. Building on the Early Learning Partnership systems research in Ethiopia (Rossiter et al., 2018), we carried out phone surveys with 480 parents and caregivers living in Addis Ababa City Administration, and primarily rural areas of Amhara, Benishangul-Gumuz, Oromia, Southern Nations, Nationalities, and Peoples’ Region (SNNPR), and Tigray. We sought to identify what learning resources and information parents and caregivers had access to during school closures, and how they were able to engage in supporting their children’s learning and wellbeing at home during school closures. Drawing on this information, this paper aims to inform the government’s short-, medium- to long-term COVID-19 response plan with respect to ECE, and to support key stakeholders in making evidence-based decisions to build the resilience of the early childhood education systems and respond adequately to any future crises.

### Risks Faced by Young Children as Consequences of Previous Crises and COVID-19

Research on the effects of previous pandemics and disasters indicates that there will be both immediate- and long-term adverse consequences, often across multiple generations. Studies tracking individuals conceived in utero, and in infancy and early childhood during pandemics and epidemics (e.g., the 1918 Spanish flu, the 1957 Asian influenza pandemic) demonstrate that those exposed can suffer life-long negative impacts, such as poor cognitive development (Kelly, 2011) and lower educational attainment (Richter & Robling, 2016). While more attention has been given to child educational outcomes associated with child survival or health, the indirect social and economic effects of the outbreak could be more severe than the outbreak itself. The outbreak of Ebola virus in west Africa in 2014, for instance, brought multi-layered socio-economic impacts, including reduced community cohesion, insecure child protection, job losses, and food insecurity (Elston et al., 2017). To date, the consequences of such crises, such as education losses and precarious home learning environment in early childhood, as well as their long-term impacts across the life course, have not been tracked widely (Benner & Mistry, 2020; Shumba et al., 2020).

In the light of the scant attention paid to early childhood development during the ongoing COVID-19 crisis, Yoshikawa et al. (2020) highlight the potentially adverse risks of COVID-19 for young children. This includes immediate impacts on their health, nutrition, care, and education, and long-term threats such as lower educational attainment and lifelong earnings, which exacerbate existing socio-economic inequalities. The authors propose mitigating actions to support early childhood development during COVID-19, which require coordinated, multi-sectoral approaches led by countries’ governments and international donors.

Lopez Boo et al. (2020) have simulated the economic costs caused by ECE closures during the pandemic across 140 high-, middle- and low-income countries. Their simulations highlight that hundreds of millions of pre-primary school-aged children are likely to suffer considerable earnings losses over their lifetimes due to the loss of early learning opportunities. Similarly, Bao et al. (2020) predict that learning disruption in the United States due to COVID-19 school closures was likely to result in a 31% reduction in reading ability gains over a nine month period in 2020. This model also predicts that reading books to young children daily at home would mitigate about 42% of the potential reduction in reading ability gains.

### Parental Support for Children’s Learning at Home

A conducive learning environment at home has been widely documented as critical for children’s acquisition of foundational skills (Chansa-Kabali et al., 2014; Dowd et al., 2017). Research suggests three dimensions of the home learning environment for early reading (see Korat et al., 2007; Van Steensel, 2006): parental education level, reading climate (i.e., the number of reading materials and children’s books available), and reading frequency (i.e., how often parents or caregivers read books to children or help their homework). Even before the pandemic, the home learning environment for children in low and lower-middle-income countries has been challenging. According to data from countries included in the latest Multiple Indicator Cluster Surveys (MICS6), disparities in the home learning environment, measured by the availability of child-oriented books and support for homework, are striking across household wealth levels, both within and across countries (Mishra et al., 2020). More than 95% of the poorest children in Iraq, Madagascar, Lesotho, Zimbabwe, and Punjab (Pakistan), for instance, live in households without any child-oriented books. This is compared with the national average that ranges between 74% in Iraq to 94% in Punjab in Pakistan (Mishra et al., 2020).

Following school closures due to COVID-19, the importance of learning at home has become even more vital. In addition to responsibilities for their children’s learning, already vulnerable households have also had to take on added responsibilities for their children’s nutrition, care, and wellbeing, which often puts considerable burden on mothers who shoulder the majority of childcare responsibilities (Gromada et al., 2020). Where parents and caregivers have had limited schooling opportunities themselves, this is likely to hinder the support they are able to provide to their children’s learning, with the high risk of perpetuating an intergenerational cycle of learning poverty.

### Ethiopian Context

In this paper, we examine the implications of COVID-19 on a sample of children from Ethiopia, a country in the Horn of Africa, with a population of more than 120 million in 2020. On the UNDP’s Human Development Index (a composite statistic based on life expectancy, education, and income per capita indicators), Ethiopia ranks near the bottom, at 173 out of 189 countries (UNDP, 2018).

Ethiopian children officially enter Grade 1 of primary school at the age of seven. Since the 1990s, Ethiopia has made remarkable progress toward achieving the Education for All goal of universal primary education. In 1992, almost four out of five children were out of school, but by 2016, the net enrolment rate reached 100% (Ministry of Education, 2016). However, despite the significant gains in enrolment, the education system continues to exhibit inequalities in access, and many children from disadvantaged backgrounds, in particular, leave school without foundational academic skills (Iyer et al., 2020).

As part of its strategy to improve educational outcomes for all children, the Ethiopian government developed a national ECE policy framework in 2010. Increasing access to ECE provision, equity, and improving ECE quality were central to this policy. Guided by this, the government has promoted the expansion of a pre-primary class, referred to as O-Class, a reception year for 6-year-olds, to improve their school readiness before they enter Grade 1. O-class has usually been accommodated within existing primary school structures, leading to an impressive increase in the gross enrolment rate of ECE children from 5.3% in 2010/11 to 46% in 2016/17 (Ministry of Education, 2010, 2017). However, this massive influx of young children into the system has created substantial challenges in the provision of equitable access to quality ECE provision in the country (Kim et al., 2021; Rossiter et al., 2018; Teferra & Hagos, 2016).

## Purpose of Study

The primary purpose of this study is to explore parents’ and caregivers’ responses to the COVID-19 crisis to ensure early learning continuity for their children during school closures. Two questions guide our analysis:

1. To what extent do parents and caregivers have access to learning resources and information during school closures due to COVID-19?
2. To what extent have parents and caregivers supported children’s learning, play, and psycho-social wellbeing at home during school closures?

## Methods

The data used in this paper were collected via phone surveys. This approach to data collection has been used previously for crisis monitoring, when face-to-face data collection is not feasible (Dabalen et al., 2016; Etang & Himelein, 2019). These phone surveys allowed us to undertake rapid, high-quality data collection from populations in various locations and settings within Ethiopia. This approach enabled us to gather the views of parents and caregivers without any threat to the safety of the fieldworkers and the participants included in the study in the context of the pandemic.

### Sampling and Participants

All participants in the 2020 phone survey had previously participated in a survey conducted in November 2019, as part of the Early Learning Partnership (ELP) research in Ethiopia. During the 2019 ELP survey, household respondents were asked to provide mobile phone numbers so that they could be contacted in the follow-up surveys if they moved from their sample location. At least one valid phone number was obtained for 1,985 out of 3,219 households (62% of households). This was the basis for the sampling frame for the 2020 phone survey. It is therefore important to note that the views captured in the phone survey are inevitably confined to parents and caregivers who possess a mobile phone. This could create some bias in responses, given that our previous analysis has shown that poorer households are less likely to possess a phone (Kim & Rose, 2020).

Urban households were also more likely to have a valid phone number than rural households: 80% and 54%, respectively. Given our interest in understanding how different population groups have been affected by school closures due to the pandemic, we adopted purposive sampling to take account of different locations (regional, and rural–urban). In addition, we purposively sampled according to children’s ECE enrolment status (to ensure a mix both of those who had attended O-Class and those who had not prior to school closures), gender, and caregivers’ literacy (measured according to whether they were able to read a sentence during the face-to-face interview conducted in November 2019).^2^ First, we selected six regions out of the original seven that were included in the 2019 ELP survey to secure a sufficient number of households in each region. Among the selected six regions, we included Addis Ababa as an urban sample. In the remaining five regions, we prioritised rural households, given their populations are predominantly rural. Through this process, the phone survey sample consisted of 76% rural and 24% urban households, which is close to Ethiopia’s average rural–urban population ratio (World Bank, 2019).

Second, we used a three-stage selection process to obtain a sample disaggregated by children’s O-class enrolment status, gender, and caregivers’ literacy. Table 1 summarizes the characteristics of the final sample. More than two-thirds of children in the sample households were enrolled in O-class. The sample is evenly distributed across children’s gender and with a similar proportion of caregivers who are and are not literate. It is important to note that the resultant sample is not intended to be representative of regions included in the phone survey.

**Table 1:** Primary caregivers and parents included in the phone survey (n = 480)

	Mean or %	SD	Min	Max
Living in rural area	76.5%	0.4		
Child ever attended ECE	77.3%	0.4		
Child is female	50.2%	0.5		
Caregiver is literate	52.1%	0.5		
Child age in years	6.7	0.7	6	11
Caregiver is female	89.6%	0.3		
Caregiver ever attended school	63.8%	0.5		

### Instrument

The phone survey drew on core modules developed by the ELP Systems Research Program at the World Bank, from which the Ethiopia ELP team selected items that were most relevant to the context. The core module included a set of items drawn from the caregiver section of the Measuring Early Learning Quality and Outcomes (MELQO) assessment. This is a globally validated, standardized assessment which is used to measure child development and learning (UNESCO et al., 2017; Raikes et al., 2019). Additional items were added to the phone survey that would help us understand the experience of families amid the COVID-19 pandemic in the Ethiopian context. Additional items were added to the phone survey that would help us understand experience of families amid the COVID-19 pandemic in the Ethiopian context, aligned with the other phone survey in Ethiopia (Yorke et al., 2021).

We predominantly included pre-coded questions in the instrument. This strategy was chosen to reach a relatively large number of respondents, with due consideration for the length of the survey. The instruments were designed to last for a maximum of 45 min, including 44 items for caregivers with children who participated in O-class, and 40 items for those with children who were not attending O-class before the pandemic. We included a varied mix of pre-coded questions, including binary questions (answering yes/no), degree questions (1–5 scales), and questions where participants had to rank responses, to allow for a wide range of information to be collected. The instruments underwent an iterative revision processes within the team, notably based in Ethiopia, to make them most relevant to the country-specific context. Given that many of our participants were likely to be in precarious circumstances, we decided not to include any questions asking sensitive information through the survey. To check the clarity of the questions, we conducted a pilot with teachers and parents living in Addis Ababa. The pilot survey confirmed that participants could understand the questions, and so no major revisions were needed. The instruments were translated into three local languages, including Amharic, Afan Oromo and Tigrigna.

Prior to conducting the phone surveys, ethical approval was obtained from the Ethical Review Board of the College of Education and Behavioural Studies at Addis Ababa University, and from the Faculty of Education at the University of Cambridge. We were particularly mindful of the disruption that COVID-19 was having on the lives of those involved in the study. All participants provided informed verbal consent. They were presented with the option to participate in the phone interview immediately, or to arrange a suitable time for the researcher to call back. Participants received compensation (100 ETB phone credit) once the interview was completed, drawing on best practice from within Ethiopia, and in line with ethically sound procedures (Morrow, 2012). All data were captured through tablets and were uploaded directly to the designed online storage system and immediately anonymised. The phone survey took 15 days to complete, and data collection and management was carried out between mid-August and September 2020.

Of the 480 households selected at the initial stage, 58 (or 12%) households were substituted with other households on the replacement list, which was prepared before data collection in case some respondents could not be reached. Where the initial sample was replaced, this was most commonly due to failure to obtain a response from the respondents, failure to acquire the correct contact details of the participants or, in a very small number of cases, because those contacted declined to participate.

In terms of response rates, about 98.5% of respondents with whom the research assistants made initial contact, agreed to participate in the research. The fact that we had previously conducted face-to-face interviews with them, is likely to have contributed to the high response rate in the current study. The majority of the calls were uninterrupted (65.6%), a few were interrupted but subsequently continued and completed (15.4%), and a very small number were interrupted and continued at a different time (0.9%). A poor network connection was often the reason for some of the interrupted calls.

### Data Analysis

For the data analysis, we merged data collected via the phone survey with the respondents’ background information collected during the ELP survey conducted in November 2019. This enabled us to analyse the data to show differences in caregivers’ COVID-19 responses across various groups of respondents (including according to urban–rural location, wealth, and caregivers’ literacy). Descriptive analysis presented in the next section was undertaken using Stata 16.

## Results

### The Economic Impact of COVID-19 on Families

Parents and caregivers have faced serious financial constraints as a result of the pandemic. The survey results show that nearly 80% percent of parents and caregivers living with pre-primary school-aged children reported that their family experienced a loss of household income since the pandemic, with poorer families being disproportionately affected (81% in poorest households compared with 71% in richest households). About four in 10 households reported that they had experienced a shortage of food since the outbreak of COVID-19, which raised concerns about insufficient food and nutrition for children. Despite the prevailing economic burden of COVID-19, less than half of households reported that they had taken some measures to try and mitigate their financial difficulties. Among those who used coping measures, two-thirds reported cutting household spending on food and non-food purchases, 16.1% received assistance from friends or families, while some received remittances (5.2%), or assistance from the government (4.2%).

### Limited Access to Learning Resources and Information During School Closures

**Fig. 1:**
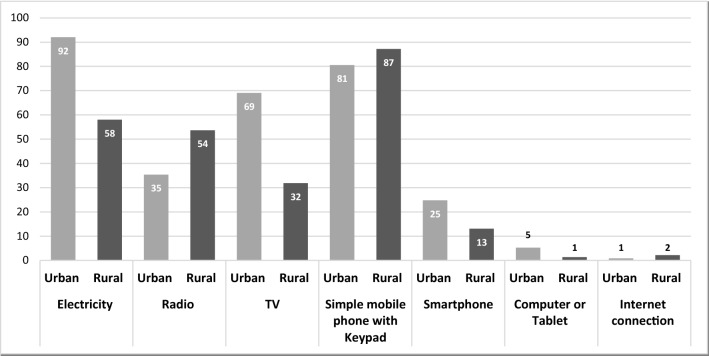
Household’s access to electricity and communication devices (%)

Our first research question addresses the extent to which parents and caregivers had access to learning resources and information during school closures due to COVID-19. Figure 1 highlights a stark difference between urban and rural families in terms of their access to electricity and communication devices to support remote learning. The vast majority of urban households have access to electricity, and most have a TV. By contrast, only around a half of rural households have access to electricity, with fewer than a third having access to a TV. Only half of rural households own a radio, although this is higher than urban households. However, around one in four urban households has access to smartphones (higher than those in rural areas) through which they might also access radio programmes. Access to a computer, tablet, or internet connection is generally very low—less than five percent—regardless of location.

**Fig. 2:**
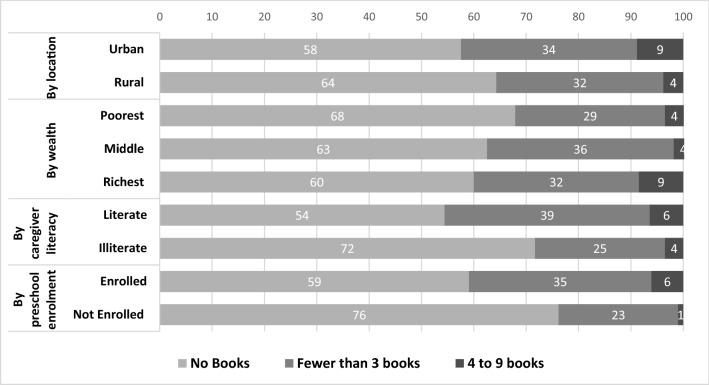
Children’s books or picture books at home (%)

Figure 2 identifies that there is uneven access to basic home learning resources such as children’s books or picture books, which can limit parents’ ability to support students, especially where there is no access to technology. More than half of parents and caregivers reported that they did not have such books at home. Notably, there is a significant gap between urban and rural households, the poorest and wealthiest households, literate and illiterate caregivers, and by children’s O-class enrolment status. Caregivers who are not literate are much less likely to have children’s books at home (72 versus 54% for no books). Children who have not enrolled in school are also much less likely to have such books at home, compared to their peers who are enrolled in O-class (76 versus 59% for no books).

This indicates that learning disruption is likely to be substantial, given that many households have limited learning resources of relevance to young children. Not surprisingly, parents reported that the biggest challenges they have faced in home-based learning included a lack of home learning materials (51.5%), followed by a lack of radio, TV, or tablet for remote education (14.2%), a lack of information and knowledge on child development (13.4%), and a lack of radio/TV educational programmes for young children (9.2%).

In addition, most parents and caregivers have received little information from schools or local governments on how to support their children’s learning during school closures. Only 10% of caregivers with children enrolled in O-class reported that they have been in contact with ECE teachers or school principals. More affluent households were more than twice as likely to be in contact with ECE teachers or school principals (15%) than poorer households (6%). Importantly, caregivers who had communicated with teachers or principals were more likely to be engaged in their child’s learning activities at home.

### Supporting Children’s Learning Activities and Play at Home During School Closures

**Fig. 3:**
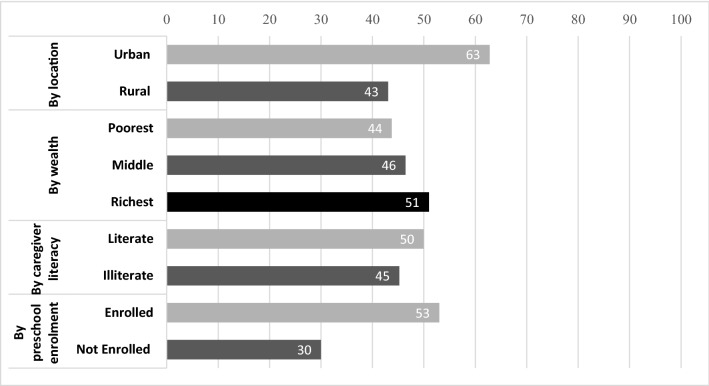
Parental engagement in child learning activities at home during school closures (%)

Our second research question assesses the extent to which parents and caregivers have been able to support their children’s learning activities and play at home during school closures. Figure 3 shows that only half of parents and caregivers reported that they had been involved in supporting educational or learning activities of their children through hiring a tutor, encouraging remote learning via radio, TV, or online learning apps, receiving assignments from teachers or caregivers, or reading books obtained from schools. Families living in urban areas, from wealthier backgrounds, and those with literate caregivers or children enrolled in O-class were more likely to engage in these activities.

As shown in Table 2, among those families who reported their involvement in home-based learning activities, more than half of parents and caregivers were likely to prepare assignments for their children to complete. For pre-primary school-aged children, these assignments might relate to activities associated with singing or storytelling, as well as those related to literacy or numeracy (such as writing or counting).

**Table 2:** Parental engagement in child learning activities and play at home during school closures (%)

	Total	Rural	Urban	Poorest	Middle	Richest	Illiterate	Literate
Engaged in educational or learning activities	47.7	43.1	62.8	43.8	46.4	51	45.2	50
(1) Session/meeting with lesson teacher	5.7	5.7	5.6	8.2	5.1	4.9	7.7	4
(2) Listened to education programmes on radio	21.4	26.6	9.9	14.3	20.5	25.5	23	20
(3) Watched educational TV programmes	16.6	13.9	22.5	8.2	2.6	31.4	14.4	18.4
(4) Used online/mobile learning apps	1.3	0.6	2.8	0	1.3	2	0	2.4
(5) Completed assignment provided by the teacher	2.6	2.5	2.8	0	3.9	3	2.9	2.4
(6) Completed assignment provided by caregivers	57.2	60.8	49.3	61.2	66.7	48	48	64.8
(7) Reading books obtained from school	28.4	17.7	52	26.5	16.7	38.2	27.9	28.8
Main responsibility of child learning and care								
(1) Mother	45.8	48	39	46.4	40	50.5	40.9	50.4
(2) Father	16.9	20	7	24.7	43.2	32.1	15.2	18.4
(3) Older siblings	25	22	35.4	19.2	38.3	42.5	30	20.4

Urban families were more likely to support their children to read books obtained from schools than rural families. Similar to the urban–rural divide in terms of access to technology, rural families helped their children to listen to educational radio programmes, while urban families helped their children to watch educational TV programmes. It should be noted that the Ethiopian government did not provide explicit radio or TV educational programmes for pre-primary school-aged children, unlike the radio programmes they provided for primary school children.

Almost half of the educational activities for pre-primary school-aged children were supported primarily by their mothers, with around one-quarter of older siblings and fewer than one in five fathers having this responsibility. Mothers in rural households were more likely to have primary responsibility for supporting child learning, even though they were less likely to be literate, and thus more likely to face challenges in supporting their children’s learning at home.

**Fig. 4:**
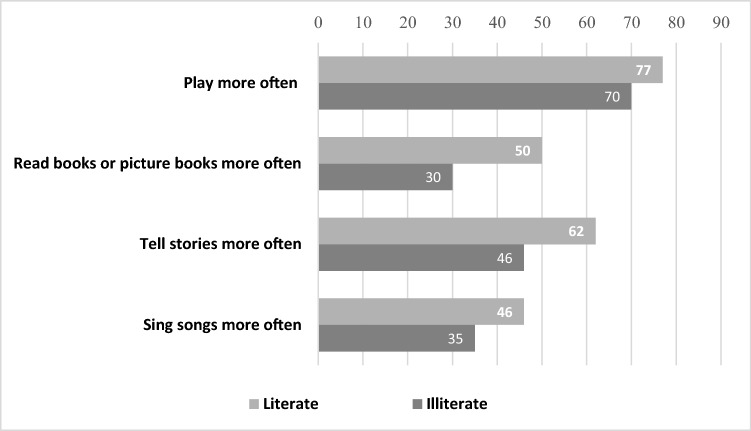
Parental support at home during school closures (%)

Positively, around three-quarters of caregivers reported that they play more often with their child since the COVID-19 crisis than they did previously, with about half of caregivers reading books, telling stories, or singing songs more often to their child while staying at home (Fig. 4). However, caregivers who are illiterate or from poorer households were less likely to play more often with their child or be involved in such activities during school closures.

### Child’s Motivation, Stress and Anxiety, and Child Discipline During School Closures

Along with families experiencing unexpected disruptions to their daily lives and welfare, some parents and caregivers also observed increased levels of stress and anxiety in their child, during school closures. Table 3 shows that about half of caregivers reported their child being less motivated in learning since schools were closed. About one-third of children cried more often since the COVID-19 pandemic began, and some were speaking less fluently or destroying or damaging things more often. However, about one-third of parents and caregivers indicated using some form of child punishment at home. Of these, nearly half reported an increased incidence of child punishment by themselves or household members since the crisis, with this increase being more apparent in households with boys, and those living in rural areas.

**Table 3:** Caregiver’s reporting on children’s motivation, behaviour and child discipline at home during school closures (%)

	Total	Rural	Urban	Poorest	Middle	Richest	Illiterate	Literate
Less motivated in learning	51.5	55.3	39	47.3	48.8	56	49.6	53.2
Crying more often	32.1	29.4	40.7	27.7	35.1	32	33	31.2
Speaking less well	18.1	16.1	24.8	12.5	18.5	21	17.4	18.8
Destroying things more often	19	19.6	16.8	20.5	20.2	17	21.3	16.8
Child punished since COVID-19	26.7	23.7	36.3	22.3	27.4	28.5	27.4	26
Caregiver								
Comforted child more often	64	62.4	69	48.2	67.9	69.5	57	70.4
Asked their feelings more often	61	57.8	71.7	48.2	63.7	66	54.8	66.8
Listened to child more often	64.4	60.5	77	52.7	65.5	70	58.7	69.6

At the same time, parents and caregivers were more likely to pay attention to their children’s psycho-social wellbeing. More than half of parents and caregivers reported that they care about children’s emotions by comforting them when s/he was feeling sad or cried, asking how the child feels, and listening to what he/she said, more often than they used to before the COVID-19 pandemic.

## Discussion

This study aimed to explore the effect of COVID-19 on children’s early learning continuity, from the perspectives of parents and caregivers. By conducting a phone survey of 480 Ethiopian households with pre-primary school-aged children between August and September 2020, we examined the extent to which parents and caregivers had access to learning resources and information during school closures caused by the COVID-19 pandemic, and the extent to which they have been able to support children’s learning, play, and wellbeing at home. In this section, we link the responses to our research questions, to existing literature and information collected in other countries that are facing similar challenges.

Our analysis shows that, in addition to the health and economic burdens from COVID-19, households have faced difficulties in supporting children’s learning at home. Parents and caregivers have had limited access to learning resources at home during school closures. Although remote learning strategies have emerged as the prominent means to ensure learning continuity, more than half of the families surveyed reported that they did not have access to either a radio or TV, with striking disparities between urban and rural, and the poorest and richest households. Moreover, despite the evidence that the availability of child-oriented books can play an important mitigating role for continued learning (Dowd et al., 2017), six in ten families reported that they did not have such books at home. This implies that children who do not have access to communication devices or reading materials at home are at high risk of being unable to continue their learning, with the poorest children being hit the hardest.

The challenges in equipping homes with learning resources are likely to be reinforced by a lack of information and support for parents from schools and governments, both of which are prevalent in Ethiopia and other low-income countries. We found that only one in ten caregivers and parents reported that they have been in contact with their child’s school. Even at the primary level, only one in five low-income countries reported providing regular phone communication between parents and schools during school closures, which has the potential to reinforce parental involvement in a child’s home learning (UNESCO et al., 2020).

Another challenge has arisen due to a lack of prioritization of ECE in policy planning within the education sector. The Ethiopian government has no remote learning policies and programmes for ECE in their COVID-19 response plan, contrary to those in primary and secondary education (Ministry of Education, 2020). This is similar to most countries worldwide: the vast majority implemented digital and broadcast remote learning policies for primary schooling, but only 60% did so for ECE (UNESCO et al., 2020). This highlights that greater attention needs to be paid by governments to prioritizing ECE in COVID-19 response planning, including with respect to remote learning programmes for young children.

Our second research question focuses on the extent to which parents supported children’s learning activities and play at home during school closures. Only about half of parents and caregivers reported that they had been involved in supporting their children’s educational or learning activities. While it is apparent that younger children require additional support from parents to engage in learning and play at home at a time of social isolation, this is unlikely to be attainable, especially for parents and caregivers who have never been to school, lower-income households, and those living in rural areas. Notably, we found that many parents have struggled to balance their responsibilities for childcare, household chores, or paid employment, with a disproportionate burden placed on mothers to support their child’s learning.

Parents and caregivers reported their efforts to create a supportive home environment, with stimulating activities such as more frequent playtime with their children, telling stories, and singing songs more often during the closure period. More than half of them also expressed how much they care about the child’s psycho-social development through these increased interactions with their children. It is important to note that the current study is confined to self-reported responses of caregivers, with a need for further research on how parents and caregivers have interacted with their children, as well as the psycho-social wellbeing of caregivers themselves. During the influenza A-H1N1 pandemic, parents who faced quarantine with limited social interactions showed higher symptoms of mental ill-health, compared to those who underwent less strict social isolation in the U.S and Canada (Sprang & Silman, 2013). Recent research indicates that more than one in six parents and caregivers in the U.S. with a 6- to 7-year-old child experienced high levels of overall stress and anxiety related to the COVID-19 pandemic (Gonzalez et al., 2020).

Moreover, a complex array of factors elevated by COVID-19—social isolation, parental stress and adversity, and uncertainty—has an impact on the psycho-social wellbeing of children. In our survey, parents often reported that their child was less motivated to learn, and expressed their stress and anxiety through more frequent crying, being less talkative, or aggressive behaviours. Some emerging evidence shows alarming patterns of children’s mental health during the spread of COVID-19. To illustrate this in other contexts, since schools were closed, children and youth were more likely to show mild to severe mental health problems such as depression, anxiety, and sleep disorders in China (Duan et al., 2020) and Bangladesh (Yeasmin et al., 2020). It is therefore critical to track the psycho-social development of young children during this pandemic, and provide appropriate measures to treat them through immediate, accessible, and affordable channels.

Although this is beyond the scope of the current study, more attention should be paid to the indirect effects of school closures, such as reduced community cohesion, domestic violence, food insecurity, and widespread job losses, which are interconnected with the disruption in early learning continuity. Our results indicate that one in six families reported an increased incidence of child punishment since school closures, particularly for boys and those living in rural areas. This highlights the importance of putting measures in place to respond to child protection risks, that are likely to be heightened in the context of the pandemic. There is also a greater risk of child malnutrition, as many school feeding programmes have been halted during the closure period, and families tend to reduce food consumption to cope with economic hardship caused by the pandemic. As shown in previous evidence from Ethiopia, malnutrition influences children’s ability to engage in education activities (Woldehanna et al., 2017). Families with young children therefore need practical support through healthcare and economic relief, such as emergency food provision, child benefits and increased cash transfers; and so also support for parental wellbeing and responsive caregiving (Yoshikawa et al., 2020), which are even more urgent in the context of the COVID-19 pandemic.

### Strengths and Limitations

This study presents important insights based on the voices of caregivers in the Global South. The focus on caregivers of pre-primary school-aged children is particularly valuable, as very little attention has been paid to the continuity of education for this age group during the COVID-19 pandemic. The study benefited from the use of globally validated measures adapted to the Ethiopian context by a team with extensive experience of undertaking research on ECE in this setting. It succeeded in achieving high response rates thanks to the team’s engagement with the respondents in a survey prior to COVID-19. Despite these strengths, there are also important limitations that should be acknowledged. First, given it was not feasible to conduct face-to-face surveys or direct observations, we were unable to collect systematic data on the types of home learning environments, and the interactions between parents and children during school closures. Such information would have allowed us to understand whether and how different home environments mitigate or exacerbate the learning loss induced by the COVID-19 school closures. Second, the study’s sample is limited to six of the 11 regions in Ethiopia. While purposive sampling allowed us to ensure our sample mirrored the national average ratio of the urban and rural population (World Bank, 2019), the findings cannot necessarily be generalized to Ethiopian children, given the diverse cultural and ethnic backgrounds of the populations. This also implies that our analysis is limited in its ability to assess the role that culture and ethnicity may have played in the way in which caregivers and families responded to the COVID-19 crisis.

## Implications and Conclusion

Schools in Ethiopia began to re-open from October 2020 on a staggered basis, after a long period of learning disruption which began in March 2020. In the absence of the government’s support for remote learning for pre-primary school-aged children, and limited parental involvement in children’s learning at home, many children are likely to have had little to no education during school closures. This is likely to have serious longer-term consequences: for example, a recent study shows that the loss of opportunity to learn due to COVID-19, including the absence of instructional time by qualified teachers, could results in a loss of 0.6 years of schooling and a reduction of $872 in yearly earnings, on average, for each student from today’s cohort in primary and secondary school (Azevedo et al., 2020).

Our findings highlight that young children and their families received very limited support from the education system in Ethiopia during school closures, especially those in poorer households and those living in rural or remote areas. Introducing strategies to promote parental involvement in children’s learning will continue to be important even after students return to school. To prevent a reinforcement of learning gaps across socio-demographic groups, these strategies need to be designed from the perspective of the challenges that parents face, including with respect to access to technology, limited availability of books and other materials in households, and a lack of guidance for parental engagement in children’s learning. Attention to the most vulnerable groups, particularly poorer households and those in which parents and caregivers are not literate, requires more comprehensive support such as the provision of cash transfers and essential supplies for families, and a strengthening of community-based platforms for parents and young children, as they are more likely to face even more challenging contexts.

Lastly, special attention needs to be paid to prioritising ECE in the government’s and aid donors’ COVID-19 response planning and financial commitment. Moreover, this public health crisis calls for coordinated response plans across health, nutrition, education, and social protection sectors to provide support for families that are particularly important in early childhood. These are critical issues that apply to Ethiopia, with important lessons for other low-income contexts facing similar challenges.

## Data Availability

Available upon request.

## Footnotes

1 For consistency, our paper used the terms “early childhood education (ECE)” to refer to any organized learning programs for children aged 3 years and above, up to the start of primary education. This covers “pre-primary education” and “preschool education” (UNICEF, 2019).

2 Data were also collected on whether the caregiver had ever attended school and the level of schooling attained. There is a strong correlation between caregivers’ literacy and having attended school (0.71, p < 0.001), and with caregivers’ level of education (0.65, p < 0.001). As such, we only retain literacy in the analysis.

## Code Availability

Stata and Microsoft Excel had been used for the analysis; Codes are available upon request.
